# Changes in circulating microRNA levels can be identified as early as day 8 of pregnancy in cattle

**DOI:** 10.1371/journal.pone.0174892

**Published:** 2017-04-05

**Authors:** Jason Ioannidis, F. Xavier Donadeu

**Affiliations:** The Roslin Institute and R(D)SVS, University of Edinburgh, Roslin, United Kingdom; University of Massachusetts Medical School, UNITED STATES

## Abstract

Poor reproductive performance remains a major issue in the dairy industry, with low conception rates having a significant impact on milk production through extended calving intervals. A major limiting factor is the lack of reliable methods for early pregnancy diagnosis. Identification of animals within a herd that fail to conceive within 3 weeks after insemination would allow early re-insemination and shorten calving intervals. In a previous study, we found an increase in plasma miR-26a levels in Day 16-pregnant relative to non-pregnant heifers, however changes in miRNA levels that early during pregnancy were very small which likely prevented the identification of robust biomarkers. In this study, we extended our analyses to a wider interval during pregnancy (Days 8 to 60, n = 11 heifers) with the rationale that this may facilitate the identification of additional early pregnancy miRNA biomarkers. Using small RNA sequencing we identified a total of 77 miRNAs that were differentially expressed on Day 60 relative to Day 0 of pregnancy. We selected 14 miRNAs for validation by RT-qPCR and confirmed significant differences in the expression of let-7f, let-7c, miR-30c, miR-101, miR-26a, miR-205 and miR-143 between Days 0 and 60. RT-qPCR profiling throughout Days 0, 8, 16 and 60 of pregnancy showed a distinct increase in circulating levels of miR-26a (3.1-fold, P = 0.046) as early as Day 8 of pregnancy. In summary, in contrast to earlier stages of pregnancy (≤ Day 24), marked differences in the levels of multiple miRNAs can be detected in circulation by Day 60 in cattle. Retrospective analyses showed miR-26a levels to be increased in circulation as early as Day 8, sooner than previously reported in any species, suggesting a biological role for this miRNA in the very early events of pregnancy.

## Introduction

Based on government estimates for 2015 [1] the UK dairy herd comprises 1.8 million cattle which produce 14.3 billion litres of milk annually (7,944 litres per animal per annum). This generates approximately £3.7 billion per annum, which is of significant importance for the UK economy overall. However, for the past 50 years the dairy industry has suffered from reduced profitability, despite a continuous net increase in milk production (which has been estimated at 1% per cow annually) [2]. In negative correlation with milk production, fertility decreased during the same period, partially as a consequence of the introduction of North American Holstein-Friesian genes in European cattle populations [2, 3]. Calving rates as low as 34% have been reported in high-producing dairy cows, with pregnancies being lost mainly during the first trimester [4]. A number of risk factors for poor reproductive performance have been identified, including immune, metabolic and endocrine parameters, with post-partum energy balance being of major importance [5]. Recently, the inclusion of reproductive traits in genetic selection programs has begun in some countries, aiming to stop the decline in reproductive performance in dairy herds, however many issues still need to be addressed [4, 6].

Key to the issue of low fertility is the inability to reliably detect early pregnancy in dairy cows, which prevents prompt re-insemination of animals failing to conceive at first service, in turn leading to extended calving intervals and suboptimal milk production [7, 8]. Current pregnancy detection methods, including trans-rectal ultrasound and palpation as well as biochemical methods such as progesterone measurements in milk, are only accurate after the first 3 weeks of pregnancy, missing out the critical window of opportunity for re-insemination of cows after failed conception, thus being inadequate to efficiently support modern intensive management systems [9].

MicroRNAs (miRNAs) are small non-coding RNAs that act as post-transcriptional gene regulators (reviewed in [10]). The intrinsically high stability of miRNAs in bio-fluids together with, in some cases [11], their tissue-specificity, confers miRNAs a significant potential as non-invasive biomarkers of disease [12–14]. So far, a plethora of studies have shown the association between specific plasma and serum miRNA signatures and human cancer, heart disease and autoimmune diseases (reviewed in [15, 16]), as well as, in a reproductive context, pregnancy progression and preeclampsia [17–19].

Despite extensive evidence in humans, the potential utility of circulating miRNAs as tissue biomarkers in livestock has not been explored widely, with a handful of recent studies identifying potential biomarkers of metabolic status and infection in chicken, pig and cow [20–22]. We recently reported for the first time changes in circulating miRNA levels during early pregnancy (Days 16 and 24) in cattle [23]. Perhaps not surprisingly, differences in miRNA levels at such early stage of pregnancy were in general very small, which, considering intrinsic biases associated with RNA sequencing [24], may have prevented the wide identification of robust miRNA biomarkers. Of note, previous studies in humans have shown that circulating miRNA profiles associated with pregnancy become more pronounced as pregnancy progresses [19]. Based on this, we followed up on our previous study by focusing on a later stage of pregnancy (Day 60) with the hypothesis that differences in levels of plasma miRNAs between Day 60-pregnant and non-pregnant (Day 0) heifers would be larger than the differences identified previously during Days 16 and 24 of pregnancy, and that this may facilitate the retrospective identification of early pregnancy biomarkers.

## Methods

### Experimental animals and sample collection

Eleven Holstein-Friesian heifers, 14–17 months old, that had never been inseminated, were oestrus-synchronised and artificially inseminated at oestrus following previously described procedures [23]. Pregnancy was confirmed by trans-rectal ultrasound on Days 35 and 60 post-insemination. Plasma samples were collected from the jugular vein in EDTA tubes (Becton Dickinson, USA) on Days 0, 8, 16 and 60 after insemination, as described before [23, 25]. The ratio between miR-451 and miR-23a was used as an indicator of haemolysis [26]; most samples had a miR ratio < 5, indicating absence of haemolysis (S1 Fig). All animal procedures were carried out under the UK Home Office Animals (Scientific Procedures) Act 1986, license 60/4604, with approval by the Ethical Review Committee, University of Edinburgh.

Cells were obtained from whole blood samples (100 μL) that were centrifuged at 1,900 g for 10 min at 4°C. The resulting supernatant was discarded and the cell pellet was re-suspended to a volume of 250 μL. The fresh blood cell solution was then used for RNA extraction as described below.

Additional body tissue samples were collected from 3–4 animals at a local abattoir and were placed in ice-cold PBS until arrival at the laboratory. Tissues were then dissected, labelled, weighed and snap-frozen using dry ice. All samples were stored at -80°C until further use.

### RNA extraction

RNA was extracted from 700 μL of plasma using TRIzol LS (Life Technologies, USA) following the manufacturer’s protocol. Following homogenisation with TRIzol LS, 3.5 μL of exogenous cel-miR-39-3p (5.6 x 10^8^ copies per sample; Qiagen, Germany) were spiked into the homogenate. Prior to RNA precipitation, 40 μg of glycogen (Sigma-Aldrich, USA) were added to each sample to facilitate precipitation of RNA in the presence of 0.1 volume of 5 M ammonium acetate salt (Sigma-Aldrich) as recommended by the manufacturer. RNA pellets were re-suspended in 20 μL of RNase-free water (Qiagen) and used immediately in downstream protocols or frozen at -80°C until further use.

Blood cell suspensions (250 μL) were homogenised using 3 volumes of TRIzol LS and extracted as per manufacturer instructions. RNA pellets were re-suspended in 10 μL of RNase-free water and frozen at -80°C until further use.

Other tissues (50 mg) were thawed in 1 mL of TRIzol LS and disrupted with Lysing Matrix D (MP Biomedicals, UK) and a FastPrep FP120 Tissue Disruptor (Thermo Electron, USA) using 2 consequent 30-second runs at a speed setting of 5. Following homogenisation, samples were centrifuged at 10,000 g for 5 min at 4°C and the resulting supernatant was aspirated. The remaining steps for RNA extraction were carried out as described in the manufacturer’s protocol and the resulting RNA pellets were re-suspended in 40 μL of RNase free water. RNA content and quality in blood-cell and tissue samples were monitored using the Nanodrop ND-1000 Spectrophotmeter (average RNA yield = 630 ng and A_260/280_ = 1.9, Thermo Fischer Scientific, USA) and *RNU6-2* expression levels.

### Small RNA sequencing

Small RNA sequencing libraries were prepared from plasma samples using the TruSeq Small RNA Library Preparation Kit (Illumina, USA) and submitted to 36-base single-end sequencing on the HiSeq 2000 Sequencing System (Illumina). Raw sequencing data (publicly available on the GEO database accession GSE83509, [27]) were analysed using the sRNAbench 1.0 tool [28, 29]. Briefly, the software was run in genome mode using default settings, with the bovine genome (bosTau4) and miRBase 21 (accessed on 30 April 2015) [30, 31] as reference in order to identify bovine miRNAs (bta-miR) and human miRNA homologues (hsa-miR). A single nucleotide mismatch was allowed when mapping raw reads to known miRNA sequences and reads without sequencing adaptor, with undetermined bases and reads below 15 nucleotides (nt) in length being automatically removed from the analysis. For additional details about the integrated analysis steps in sRNAbench please refer to the software manual.

Normalised reads (reads per million mapped, RPMM) obtained from sRNAbench were filtered before being passed to edgeR 3.10.2 for differential expression analysis in R language 3.2.1 [32, 33]. Prior to analysis, miRNAs which were detected with less than 25 RPMM in more than 8 samples per experimental group were excluded to reduce technical noise. Differential expression analysis was performed using edgeR’s paired mode on 197 miRNAs (S1 File) using GLMfit. The statistical significance threshold was set to false discovery rate (FDR) = 0.05.

### RT-qPCR

For plasma samples, cDNA was generated using the miScript II RT Kit (Qiagen) with 2 μL of RNA extract used in each 10 μL reaction. For blood cell and tissue samples, 500 ng of RNA were used in each 10 μL reaction. A Whatman-Biometra Thermocycler (Biometra, USA) was used for the reverse transcription reaction using the conditions recommended by the manufacturer.

The cDNA template was diluted 40-fold and added to 10 μL qPCR reactions that were prepared in 96-well format using the miScript SYBR Green PCR Kit (Qiagen). Template amplification was carried out using commercial species-specific miRNA primers (Qiagen) in an Agilent MX3000P qPCR system (Agilent Technologies, USA), following the cycling conditions suggested by the manufacturer. No-reverse transcriptase and no-template controls were used for each plate. Raw fluorescence data were collected using MxPro software (Agilent Technologies). The amplification efficiency of different primers ranged between 81% and 115%, with R^2^ > 0.91. Expression levels were determined using freshly-made standard curves and data were analysed further using Microsoft Excel (Microsoft Corporation, USA). For plasma samples, expression levels were normalised to the mean expression of cel-miR-39-3p and miR-128 (miR-128 was the miRNA with the least variation across samples with coefficient of variation (CV) = 15%). For blood cells and tissues, expression data were normalised to *RNU6-2*. Samples with distinctly low *RNU6-2* levels were excluded.

Statistical analyses were carried out using GraphPad Prism 7 (GraphPad Software, USA). Differences in miRNA expression between Day 0 and Day 60 were assessed using paired t-tests. For data involving more than 2 time points one-way rmANOVA was used to determine the effect of Day, followed by Dunnett’s test to compare each time point with the Day 0 control. In all cases, normality was tested using the Shapiro-Wilk normality test, outliers were identified with the ROUT test and then removed, and data were log_2_(x+1) transformed to meet the tests’ normality criteria. Statistical significance thresholds for all tests were set to P = 0.05.

### Genomic cluster and pathway analyses

Information on genomic miRNA clusters in cow was obtained from miRBase 21 (accessed 23/06/2016) [31]. Genomic clusters were numbered in order of decreasing size as shown on miRBase, and the overlap with our sequencing gene list was identified manually.

Target and pathway analysis was carried out using DIANA miRPath 3.0 [34]. Experimentally validated miRNA targets were identified using DIANA-TarBase 7.0 and the Pathway Union option (an *a posteriori* method) was used for pathway analysis. The integrated Fischer’s exact test followed by FDR adjustment were used for statistical analysis. Enriched pathways from the KEGG database were exported from the tool along with the corresponding FDR-corrected P values. A significance threshold of FDR = 0.05 was applied to the corrected P values.

## Results and discussion

### Small RNA sequencing of bovine plasma

**Fig 1:**
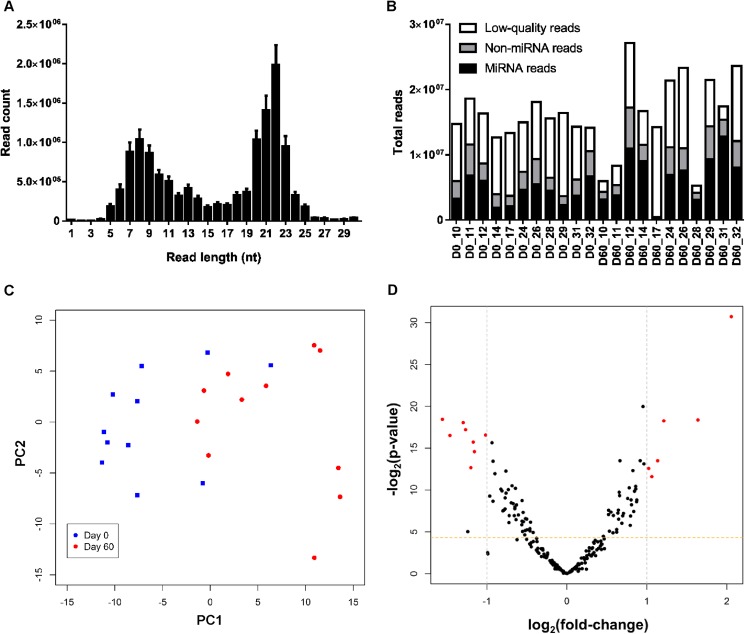
Small RNA sequencing results. (A) Read length (mean ± SEM) distribution plot for all samples constructed using raw reads. (B) Barplot showing total, mapped and miRNA reads for each plasma sample. Sample P60_17 generated very few miRNA reads, however it was included because it clustered normally in subsequent principal component analysis (PCA). (C) PCA plot using transformed normalised data and showing partial separation between Day 0 and Day 60 samples. (D) Volcano plot showing differences in individual miRNA values between Days 0 and 60 of pregnancy, using transformed normalised data. Values with fold-change > 2 and FDR < 0.05 are highlighted in red.

We sequenced a total of 22 plasma samples from Days 0 and 60 of pregnancy. On average, we generated 16.1 million raw reads per sample (Table 1). There was a distinct peak in read lengths between 20–23 nt, which matches the length of most mature miRNAs (Fig 1A). Out of the total raw reads, 5.6 million (34.7%) corresponded to bovine and human mature miRNA sequences (Table 1, Fig 1B). Across all samples, we identified up to 389 unique miRNAs present at more than 10 reads. The 15 most abundant miRNAs in bovine plasma are listed in Table 2. Eleven of these miRNAs were also among the most abundant in our previous cattle study [23].

**Table 1:** Mean numbers of reads from sequencing of plasma samples on Days 0 and 60 of pregnancy (n = 11 heifers), including reads which were excluded from further analyses.

	Day 0	Day 60	Mean
Raw reads	15,382,609	16,807,294	16,094,951
Reads without adapter (excluded)	120,529	143,187	131,858
Short reads < 15 nt (excluded)	8,185,927	6,897,753	7,541,840
Low quality reads (excluded)	23,372	26,179	24,775
Reads mapping to bovine genome	7,052,780	9,740,175	8,396,478
Bovine miRNA reads	4,322,232	6,814,346	5,568,289
Bovine miRNAs detected at > 10 reads (range)	268–337	193–353	N/A
Human homologous miRNA reads	7,469	5,451	6,460
Human miRNAs detected at > 10 reads (range)	18–32	5–36	N/A

**Table 2:** Most abundant miRNAs (RPMM) in bovine plasma on Days 0 and 60 of pregnancy (n = 11 heifers) identified by sequencing.

miRNA	Day 0	Day 60	Mean
bta-miR-486	163,304	187,148	175,226
bta-miR-22-3p	115,215	86,953	101,084
bta-miR-192	55,994	45,163	50,578
bta-miR-27b	50,069	39,846	44,957
bta-miR-143	16,617	67,181	41,899
bta-miR-10b	49,529	30,810	40,169
bta-miR-142-5p	31,254	40,063	35,659
bta-miR-191	32,881	33,804	33,343
bta-miR-423-5p	34,453	23,760	29,106
bta-miR-451	19,021	27,249	23,135
bta-miR-21-5p	21,387	24,164	22,775
bta-miR-101	17,776	25,853	21,814
bta-miR-92a	23,371	19,306	21,339
bta-miR-103	27,287	13,565	20,426
bta-miR-30d	17,498	18,882	18,190

From our comprehensive miRNA profiling dataset we sought to identify stable miRNAs which could be utilised for data normalisation in subsequent steps. The miRNA with the lowest variability across samples was miR-128 (CV = 15%) followed by miR-151-3p and miR-425-5p (Table 3). This result was confirmed by NormFinder, which listed miR-128 as the second least variable miRNA in the dataset after miR-181b, with stability values of 0.07 and 0.06, respectively (S1 File, Table 3). We choose miR-128 as an endogenous normaliser for subsequent RT-qPCR assays over miR-181b on the basis of its higher abundance (see ‘Methods‘).

**Table 3:** The 20 most stable miRNAs in bovine plasma from Days 0 and 60 of pregnancy identified using sequencing (n = 22 samples).

miRNA	RPMM	Coefficient of variation	NormFinder stability value
bta-miR-128	2,687	15%	0.07
bta-miR-151-3p	6,732	16%	0.12
bta-miR-425-5p	1,724	17%	0.07
bta-miR-1307	266	20%	0.08
bta-miR-181b	1,295	21%	0.06
bta-miR-148a	15,092	21%	0.09
bta-miR-130b	1,085	21%	0.12
bta-miR-148b	351	21%	0.13
bta-miR-181a	13,983	22%	0.11
bta-miR-1842	113	23%	0.11
bta-miR-6119-3p	40	23%	0.12
bta-miR-92a	21,339	24%	0.13
bta-miR-30a-5p	6,808	26%	0.12
bta-miR-27b	44,957	27%	0.13
bta-miR-29c	733	27%	0.12
bta-miR-151-5p	1,667	27%	0.13
bta-miR-326	174	30%	0.13
bta-miR-744	318	31%	0.14
bta-miR-194	442	33%	0.13
bta-miR-224	86	37%	0.14

### Identification of differentially expressed miRNAs on Day 60 of pregnancy

Principal component analysis (PCA) revealed partial separation of samples based on Day of pregnancy as shown in Fig 1C. Differential expression analysis identified 77 miRNAs which were differentially expressed on Day 60 compared to Day 0 with FDR < 0.05 (Fig 1D, Table 4; S1 File). Fourteen miRNAs changed by more than 2-fold between groups and the largest difference was observed for let-7c, which increased 4.5-fold on Day 60 of pregnancy.

**Table 4:** Top differentially expressed miRNAs in plasma between Day 0 and 60 of pregnancy (n = 11 heifers).

miRNA	Day 0*	Day 60*	Fold-change	FDR
bta-let-7c	273	932	4.53	0.000
bta-miR-143	16617	67181	4.11	0.000
bta-miR-16a	1965	4212	2.68	0.048
bta-miR-155	250	477	2.32	0.000
bta-miR-19a	298	569	2.28	0.001
bta-miR-19b	1036	1913	2.13	0.003
bta-let-7f	4265	6502	2.12	0.012
bta-miR-32	387	792	2.01	0.002
bta-miR-374b	71	112	2.00	0.007
bta-miR-26a	9042	13806	1.95	0.006
bta-miR-26b	2971	4506	1.94	0.011
bta-miR-324	41	18	0.51	0.001
bta-miR-665	103	45	0.51	0.000
bta-miR-410	267	124	0.50	0.000
bta-miR-328	143	54	0.49	0.000
bta-miR-23a	7123	2795	0.49	0.000
bta-miR-99b	1142	414	0.49	0.002
bta-miR-424-3p	32	11	0.47	0.000
bta-miR-205	1072	344	0.46	0.000
hsa-miR-4532	945	229	0.42	0.000

*Values shown for Days 0 and 60 are mean RPMMs. Note that miR-4532 is a novel human homologue not annotated in cow in miRBase 21.

Given the identification of miRNA clusters associated with pregnancy in other species [35], we next sought to determine whether the miRNAs differentially expressed between Days 0 and 60 of pregnancy belonged to specific genomic clusters. A total of 82 miRNA clusters, each containing 2 to 47 miRNAs, are registered for cow in miRBase 21. Using this as reference, we found that 31 out of the 77 differentially expressed miRNAs belonged to a cluster, being present together with at least another miRNA from the same cluster (Table 5). In total, 13 miRNA clusters were represented among differentially expressed miRNAs. Among these were the miR-379~656 cluster (known as C14MC in humans), represented by 5 miRNAs, and the miR-17~92 and miR-106b~25 clusters, represented by 5 and 2 miRNAs, respectively (Table 5). The C14MC cluster is known to be expressed in the placenta and is involved in foetal development; circulating levels of C14MC miRNAs increase during human pregnancy [36, 19, 37, 38]. The miR-17~92 and miR-106b~25 clusters are paralogues, with a demonstrated role in vascular endothelial growth factor (VEGF)-mediated angiogenesis through targeting of phosphatase and tensin homolog (PTEN), and a possible role in placental immune tolerance during the first trimester of pregnancy; both clusters are highly expressed in the human placenta [36, 39–41]. Finally, several members of the let-7 family known to be expressed in the human placenta [36] were present across multiple genomic clusters (Table 5). Overall, these results suggest functionally conserved roles of a subset of pregnancy-associated miRNA clusters across species.

**Table 5:** Genomic clusters represented by miRNAs differentially expressed in plasma between Days 0 and 60 of pregnancy (n = 11 heifers).

Cluster*	Genomic location	No. of miRNAs in cluster	Differentially expressed miRNAs between Days 0 and 60 of pregnancy
miR-379~656	chr21:67561869–67604767	47	mir-380, mir-154c, mir-381, mir-369, mir-410
miR-424~450b	chrX:18185439–18178985	7	mir-424, mir-450a-2, mir-450a-1, mir-450b
miR-493~136	chr21:67416605–67431509	7	mir-665, mir-136
miR-17~92	chr12:66226554–66227332	6	mir-17, mir-18a, mir-19a, mir-20a, mir-19b
let-7a-3~3596	chr5:117119385–117120270	4	let-7a-3, let-7b
let-7a-1~7d	chr8:86884872–86887517	3	let-7a-1, let-7f-1
miR-106b~25	chr25:36892046–36892532	3	mir-106b, mir-93
miR-199a~214	chr16:40491602–40485916	3	mir-199a-1, mir-214
miR-23a~24	chr7:12981970–12981713	3	mir-23a, mir-27a
miR-452~3431	chrX:34665624–34662756	3	mir-452, mir-224, mir-3431
miR-99b~125b	chr18:58014868–58015620	3	mir-99b, mir-125a
miR-100~let-7a-2	chr15:33353395–33347640	2	mir-100, let-7a-2
let-7-2~miR-98	chrX:96383532–96382746	2	let-7f-2, mir-98

*Only clusters for which at least 2 miRNAs were differentially expressed are shown

To gain insight on the roles of the differentially expressed miRNAs during pregnancy we carried out pathway enrichment analysis, using the DIANA web-tool [34], separately using experimentally validated targets of all the up-regulated and down-regulated miRNAs on Day 60 of pregnancy (39,555 and 17,439 targets, respectively). There was significant overlap between enriched pathways targeted by up-regulated and down-regulated miRNAs (S2 File), and these common pathways included fatty acid synthesis and metabolism, extracellular cell membrane (ECM)-receptor interaction, adherens junction, Hippo signalling and the thyroid hormone, p53 and cell cycle pathways. Up-regulated miRNAs in pregnancy also targeted the forkhead O (FOXO) and transforming growth factor beta (TGF-β) signalling pathways. These results suggest roles of miRNAs in regulating cell metabolism as well as proliferation/survival/migration processes involved in tissue growth during pregnancy.

### Validation of sequencing data using RT-qPCR

**Fig 2:**
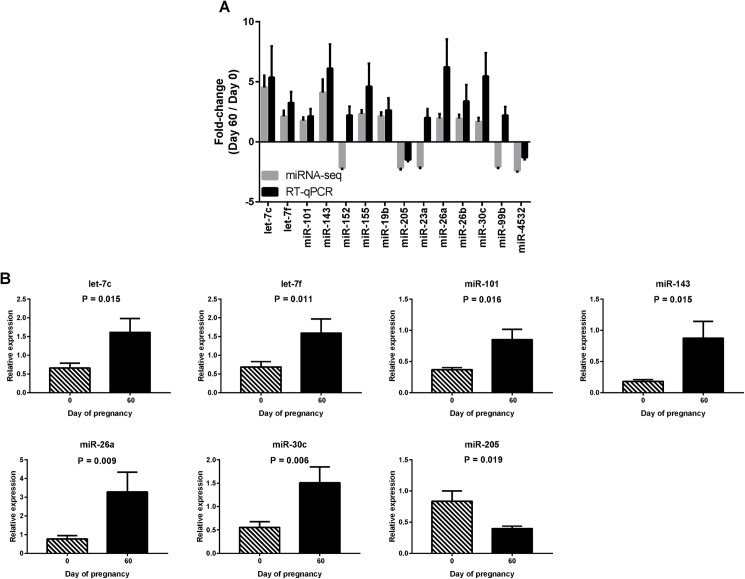
RT-qPCR validation of selected sequencing results. (A) Fold-changes in plasma miRNA levels (mean ± SEM) identified during pregnancy using sequencing and RT-qPCR. (B) Relative expression (mean ± SEM, normalised to the mean expression of cel-miR-39-3p and miR-128) of 7 miRNAs on Days 0 and 60 of pregnancy obtained by RT-qPCR.

Previous studies with human tissues have shown only partial agreement in miRNA expression data generated using different profiling platforms [42]. Thus, we set out to validate our results from sequencing using RT-qPCR. Fourteen differentially expressed miRNAs were selected for validation (Table 6) based on both their relative abundance (RPMM) and fold-change between Days 0 and 60 (Table 4, S1 File). For 11 out of the 14 miRNAs, RT-qPCR results agreed with those from sequencing (Table 6, Fig 2A), although statistical significance was obtained only for 7 miRNAs, namely let-7c, -7f, miR-101, -30c, -26a, -143 and -205. Six miRNAs were expressed at significantly higher levels (up to 6-fold, P < 0.05) on Day 60 compared to Day 0, while miR-205 was significantly lower on Day 60 (1.5-fold, P < 0.05, Fig 2B, Table 6). Our subsequent analyses focused on these 7 miRNAs.

**Table 6:** Differences in expression of plasma miRNAs between Days 0 and Day 60 of pregnancy (n = 11 heifers) obtained by sequencing and RT-qPCR.

	Next-generation sequencing			RT-qPCR	
miRNA	Fold-change	P value	FDR	Fold-change	P value
bta-let-7c	4.53	0.000	0.000	5.30	0.015
bta-miR-143	4.11	0.000	0.000	6.11	0.015
bta-miR-155	2.32	0.000	0.000	4.60	0.053
bta-miR-19b	2.13	0.000	0.003	2.63	0.929
bta-let-7f	2.12	0.003	0.012	3.25	0.011
bta-miR-26a	1.95	0.001	0.006	6.22	0.009
bta-miR-26b	1.94	0.002	0.011	3.38	0.054
bta-miR-101	1.76	0.003	0.014	2.12	0.016
bta-miR-30c	1.67	0.032	0.075	5.50	0.006
bta-miR-23a	0.49	0.000	0.000	2.00	0.312
bta-miR-99b	0.49	0.000	0.002	2.20	0.687
bta-miR-152	0.46	0.031	0.073	2.21	0.869
bta-miR-205	0.46	0.000	0.000	0.67	0.012
hsa-miR-4532	0.42	0.000	0.000	0.77	0.161

Potential roles for these miRNAs during pregnancy can be speculated based on previous literature and the results of gene ontology analyses above (S2 File). Four of the seven miRNAs, miR-143, miR-101, miR-30c and miR-26a, have been shown in previous studies to regulate insulin signalling and lipid metabolism and to be involved in diabetes [43–46], and thus they may play important roles regulating changes in maternal metabolism occurring early during pregnancy. In addition, miR-26a and let-7f have been shown to regulate macrophage- and T-cell-mediated immunity [47–50] which would be consistent with a role in establishing maternal immuno-tolerance, a key function during early pregnancy. Finally, miR-101, miR-205 and miR-26a are known regulators of angiogenesis [51–54], a key process during development of foeto-placental and maternal tissues during pregnancy.

### Tissue-wide expression of plasma miRNAs associated with pregnancy

**Fig 3:**
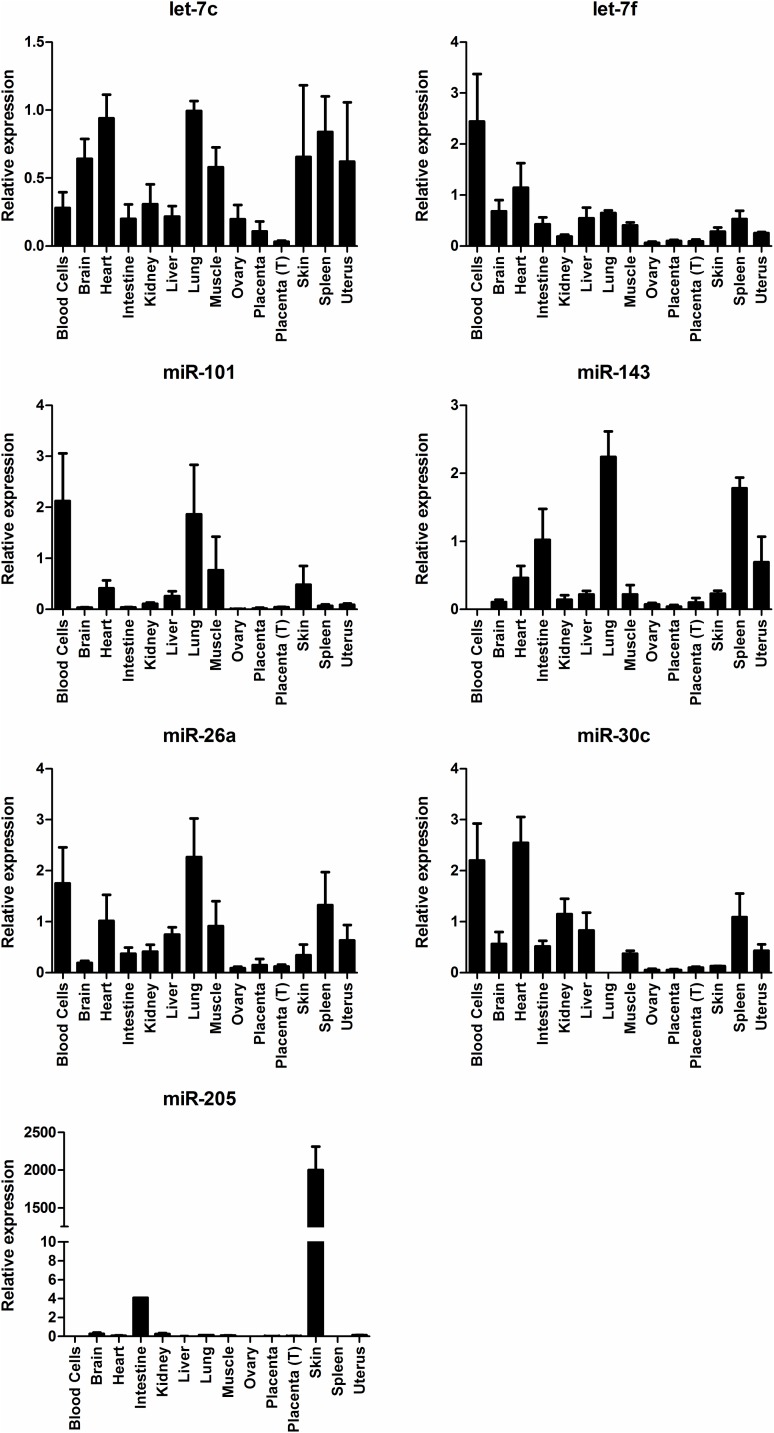
Expression of pregnancy-associated miRNAs in different tissues. Relative expression (mean ± SEM, normalised to *RNU6-2* expression) of pregnancy-associated miRNAs in 14 tissues from healthy cows (n = 3 animals per tissue). Placenta: Placenta from Day 70 of pregnancy; Placenta (T): Term placenta.

Next, to investigate the origin of these miRNAs, and at the same time gain further insight on their potential roles during early pregnancy, we profiled the expression of all 7 miRNAs across 14 different bovine tissues, including blood cells. A total of 4 outliers were identified and removed from each of let-7f, let-7c, miR-101 and miR-143 datasets. Six miRNAs (let-7c, let-7f, miR-101, miR-143, miR-30c and miR-26a) were expressed in many different body tissues including the uterus and the placenta, with no particular tissue being clearly enriched for any particular miRNA (Fig 3), overall consistent with data from human tissues [55, 56]. Notably, relative expression levels for most miRNAs were lowest in both early (Day 70) and term placenta, indicating the developing conceptus may not be a significant source of these miRNAs in plasma. In addition, expression profiles suggested that, for all miRNAs except miR-143 and miR-205, blood cells are a significant source of plasma levels, consistent with roles in immuno-regulation as suggested above. Interestingly, mean levels of miR-205 were more than 8,000-fold higher in skin than in any other tissue (Fig 3). In mammals, miR-205 was reportedly expressed in a range of tissues including the thymus, mammary gland, early embryo, digestive tissue and skin [57–59, 55], however, different studies highlighted the importance of miR-205 in regulating keratinocyte function [60, 61], which is consistent with our results. Whether skin is indeed a significant contributor to circulating levels of miR-205 should be explored in future studies.

A caveat associated with our tissue profiling approach is that all tissues analysed (other than placenta) were collected from non-pregnant animals, which would not account for changes in tissue miRNA expression that may only occur during pregnancy. However, since our goal was to determine whether any of the miRNAs analysed (Fig 3) was specifically expressed or highly enriched in a particular tissue, which we can reasonably assume will not depend on pregnancy status, then the use of tissues from non-pregnant heifers for profiling those miRNAs was justified.

### Profiling of circulating miRNAs throughout early pregnancy

**Fig 4:**
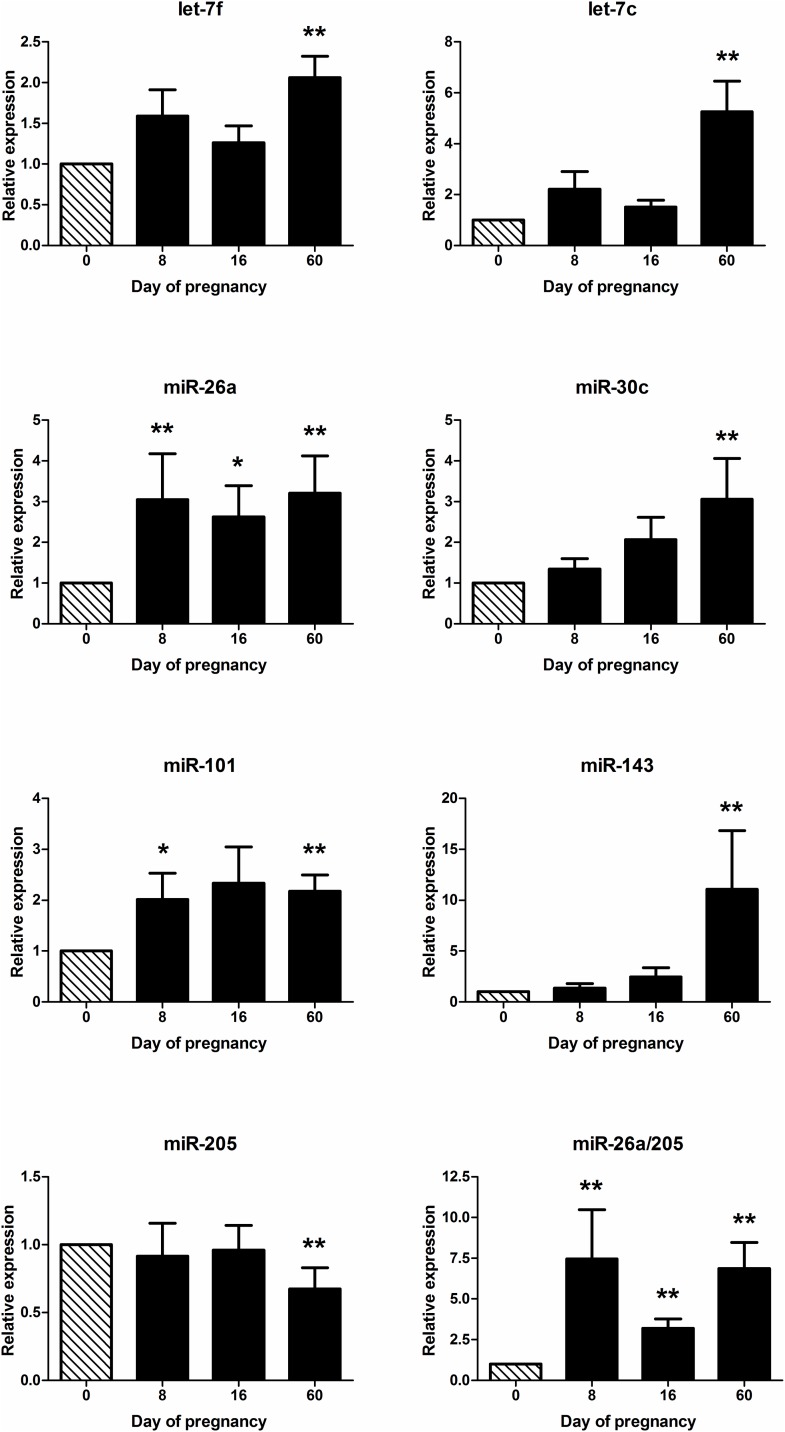
MiRNA profiles throughout early pregnancy determined by RT-qPCR. Relative expression (mean ± SEM, normalised to the mean expression of cel-miR-39-3p and miR-128) of selected miRNAs in plasma during Days 0, 8, 16 and 60 of pregnancy. For clarity, fold changes in miRNA expression relative to Day 0 are shown. Day means with one or two asterisks are different (P < 0.1 and P < 0.05, respectively) from the corresponding mean on Day 0.

Finally, we sought to obtain further insight on these pregnancy-associated miRNAs by determining their temporal changes in circulation throughout early pregnancy. We hypothesised that some of the miRNAs identified on Day 60 may actually begin increasing at earlier stages of pregnancy (at or before Day 16), which could provide useful biomarkers of early pregnancy, possibly in the form of a miRNA panel. Accordingly, we profiled all 7 miRNAs by RT-qPCR on sequential plasma samples collected on Days 0, 8, 16 and 60 of pregnancy. We identified and removed one outlier value for each of miR-101, miR-26a and let-7f on Day 8, and miR-19a on Day 0. Analyses of the remaining data showed a significant effect of Day of pregnancy (P < 0.05) for all 7 miRNAs (Fig 4). Moreover, mean levels of many miRNAs did not change significantly until Day 60 (P < 0.05), with some miRNAs increasing gradually (non-significantly) throughout early pregnancy, e.g. miR-30c. Remarkably, however, mean levels of miR-26a were distinctly increased as early as Day 8 (3.1-fold; P = 0.046), resulting from a net increase in miR-26a levels (ranging from 1.3- to 11.7-fold) in 8 of 10 animals between Days 0 and 8. In addition, levels of miR-101 tended to increase (2.3-fold, P = 0.069) on Day 16 relative to Day 8 with no further elevation in mean levels of that miRNA up to Day 60 (Fig 4). Changes in miRNA levels in circulation this early during pregnancy (i.e., Day 8) have not been reported before and suggest a novel role for miR-26a, possibly through its immunomodulatory effects [48, 49], very early during the establishment of pregnancy, a proposition that clearly deserves further investigation. Although miR-26 has also been shown to play different roles presumably unrelated to pregnancy [62–64], the robust (3-fold) increase in plasma miR-26 levels between Days 8 and 0 of pregnancy suggest that miR-26a could be potentially used as a diagnostic biomarker as early as the first week of pregnancy, which could significantly facilitate reproductive management of modern dairy herds. Lastly, because changes in miR-26a and miR-205 showed opposite trends during early pregnancy we calculated the ratio between these 2 miRNAs to determine whether this could provide an improved diagnostic biomarker; we found the miR-26a / 205 ratio to increase 7.5-fold (P < 0.001) between Days 0 and 8 of pregnancy, providing an even more robust, potential molecular indicator of very early pregnancy in cattle (Fig 4).

## Conclusions

Our novel results using small RNA sequencing and RT-qPCR profiling show wide changes in circulating miRNA profiles occurring by Day 60 of pregnancy in cattle, in contrast with the much smaller differences previously reported from high-throughput analysis of small RNAs earlier during pregnancy (Days 16 and 24) [23]. Specifically, we identify a subset of miRNAs (let-7f, let-7c, miR-30c, miR-101, miR-26a, miR-205 and miR-143), the levels of which increase distinctly in circulation (up to 6-fold) in Day 60 pregnant relative to non-pregnant (Day 0) cows, and which provide novel molecular candidates involved in the establishment of pregnancy in cattle. Significantly, an increase in plasma levels of miR-26a was identified as early as Day 8, much earlier than previously reported for any miRNA during pregnancy in any species, suggesting one of the biological role of this miRNA may be in the very initial stages of pregnancy, providing a potential unique diagnostic biomarker of early pregnancy. Overall, the subset of miRNAs identified in our study provide the basis to elucidate specific roles of miRNAs during early pregnancy and for more comprehensive biomarker identification studies in the future.

## Supporting information

S1 Fig Haemolysis screen of plasma samples. Ratio between miR-451 and miR-23a in all plasma samples from Days 0, 8, 16 and 60 of pregnancy, as an indicator of haemolysis.
(TIF)

S1 File Sequencing miRNA profiles. Normalised expression for all miRNAs used in differential expression analysis.
(XLSX)

S2 File Pathway analysis. Pathways identified to be targeted by miRNAs that were up-regulated or down-regulated in plasma between Days 0 and 60 of pregnancy.
(XLSX)

## Abbreviations

miRNA: microRNA
nt: nucleotide
PCA: principal component analysis
qPCR: quantitative polymerase chain reaction
RPMM: reads per million mapped
RT: reverse transcription
