# Supplementary figures and images for: Changes in circulating microRNA levels can be identified as early as day 8 of pregnancy in cattle
Source: PLoS One. 2017 Apr 5;12(4):e0174892. doi: 10.1371/journal.pone.0174892 (PMC5381804; doi:10.1371/journal.pone.0174892)

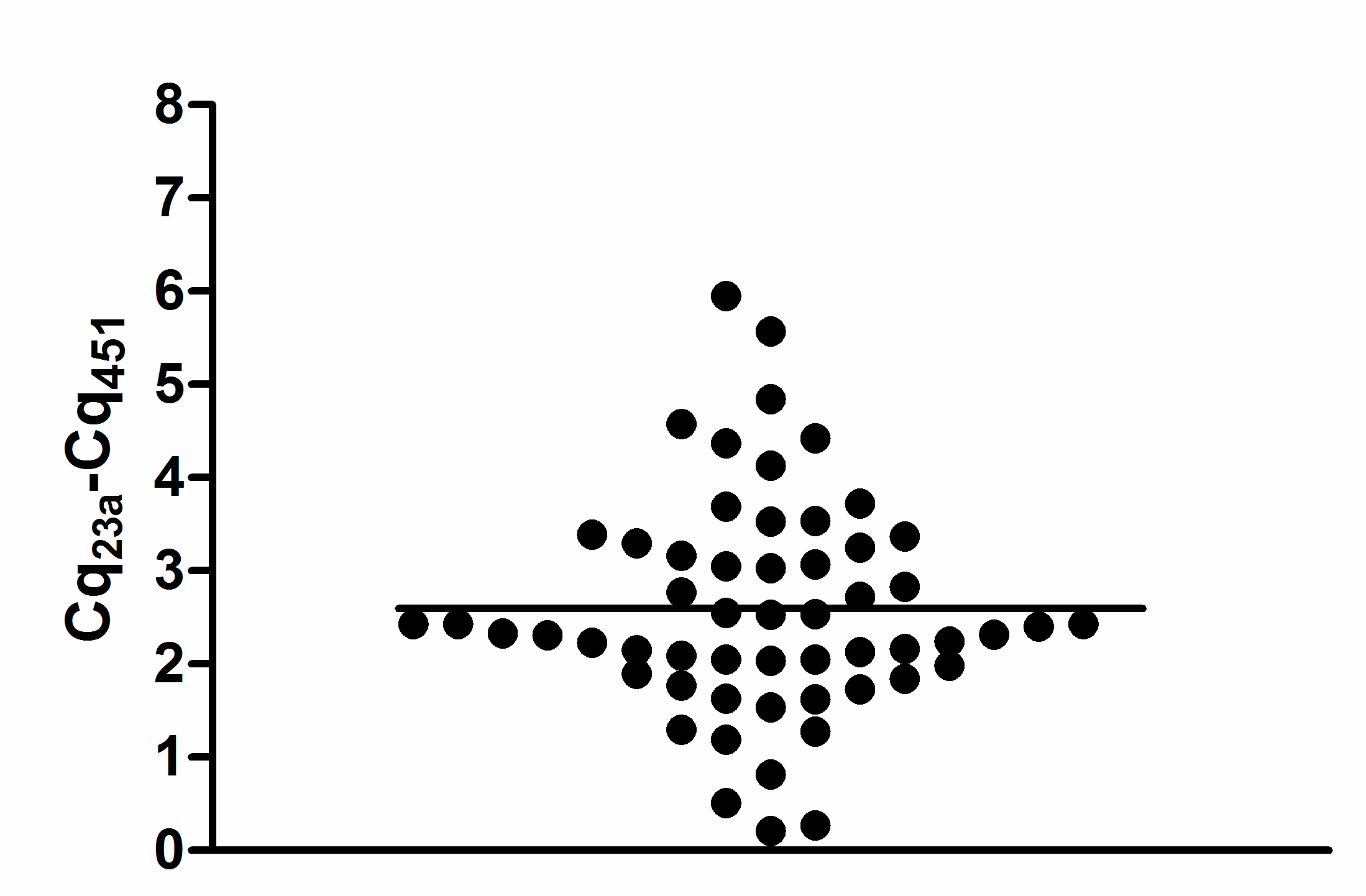

Supplement: S1 Fig — Ratio between miR-451 and miR-23a in all plasma samples from Days 0, 8, 16 and 60 of pregnancy, as an indicator of haemolysis. (TIF) [file pone.0174892.s001.tif]
